# Supplementary material for: Assessing the Pathogenicity of Two Bacteria Isolated from the Entomopathogenic Nematode Heterorhabditis indica against Galleria mellonella and Some Pest Insects
Source: Insects. 2019 Mar 26;10(3):83. doi: 10.3390/insects10030083 (PMC6468454; doi:10.3390/insects10030083)
Supplement: Supplementary file 1 [file insects-10-00083-s001.pdf]

insects

MDPI

Article

Assessing the Pathogenicity of Two Bacteria Isolated from the Entomopathogenic Nematode *Heterorhabditis indica* against *Galleria mellonella* and Some Pest Insects

Rosalba Salgado-Morales [1,2], Fernando Martínez-Ocampo [2], Verónica Obregón-Barboza [2], Kathia Vilchis-Martínez [3], Alfredo Jiménez-Pérez [3] and Edgar Dantán-González [2,*]

[1] Doctorado en Ciencias, Instituto de Investigación en Ciencias Básicas y Aplicadas, Universidad Autónoma del Estado de Morelos, Av. Universidad 1001, Chamilpa, 62209 Cuernavaca, Morelos, México; salgadomoralesr@hotmail.com

[2] Laboratorio de Estudios Ecogenómicos, Centro de Investigación en Biotecnología, Universidad Autónoma del Estado de Morelos, Av. Universidad 1001, Chamilpa, 62209 Cuernavaca, Morelos, México; fernando.martinezo@uaem.mx (F.M.-O.); veronica.obregon@uaem.mx (V.O.-B.)

[3] Centro de Desarrollo de Productos Bióticos, Instituto Politécnico Nacional, Calle Ceprobi No. 8, San Isidro, Yautepec, 62739 Morelos, México; vilchisk78@gmail.com (K.V.-M.); aljimenez@ipn.mx (A.J.-P.)

* Correspondence: edantan@uaem.mx; Tel.: +52-777-329-7000

Received: 20 December 2018; Accepted: 15 March 2019; Published: date

insects

MDPI

Supplementary Materials

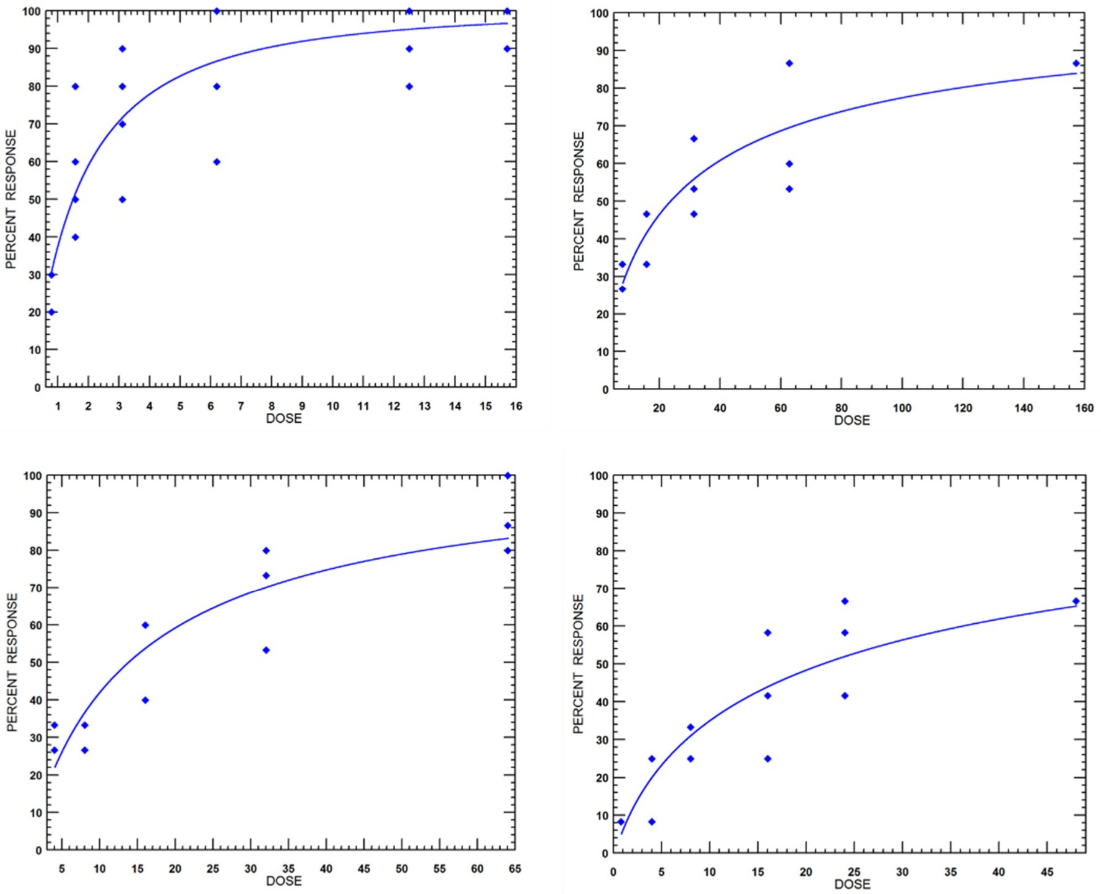

Figure 1. Dose–response curves and LC50 of *H. indica* MOR03 against insects. (**a**) *G. mellonella* LC50 = 1.4 (1.0–1.9) 95% CI) **IJs/cm²**; (**b**) *T. molitor* LC50 = 23.5 (15.8–33.3) 95% CI) **IJs/cm²**; (**c**) *H. subflexa* LC50 =13.7 (10.1–18.3) 95% CI) **IJs/cm²**; and (**d**) *D. magnifactella* LC50 = 21.7 (14.4–43.8) 95% CI) **IJs/cm²**.

Table S1. Proteins involved in the pathogenesis *Photorhabdus luminescens* HIM3.

Start	Stop	Strand	Funtion
403112	403930	+	Type III secretion thermoregulatory protein
412474	413406	+	Type III secretion injected virulence protein
88772	92383	+	Virulence sensor protein bvgS precursor
74364	75290	+	Virulence factor VirK
128132	127134	-	Type III secretion injected virulence protein YopT
2380	2592	+	Unknown, probable insecticidal toxin
2940	2686	-	YoeB toxin protein
3194	2940	-	YefM protein (antitoxin to YoeB)
4449	14645	+	RTX toxins determinant A and related Ca2+-binding proteins
16367	16603	+	CcdA protein (antitoxin to CcdB)
16606	16920	+	CcdB toxin protein
105357	101491	-	Unknown, probable toxin
54874	54656	-	CcdA protein (antitoxin to CcdB)
34760	35014	+	RelB/StbD replicon stabilization protein (antitoxin to RelE/StbE)

insects MDPI

35004	35294	+	RelE/StbE replicon stabilization toxin
36628	36449	-	ParE toxin protein
37017	36748	-	RelB/StbD replicon stabilization protein (antitoxin to RelE/StbE)
47217	47498	+	HigB toxin protein
47509	47787	+	HigA protein (antitoxin to HigB)
54294	54704	+	Antitoxin 1
82143	77728	-	Unknown, probable insecticidal toxin
85080	82192	-	Unknown, probable insecticidal toxin
11597	11295	-	FIG045511: hypothetical antitoxin (to FIG022160: hypothetical toxin)
11893	11594	-	FIG022160: hypothetical toxin
70910	68766	-	RTX toxin transporter, ATP-binding protein
72268	70913	-	RTX toxin transporter, determinant D
74366	72261	-	RTX toxin transporter, ATP-binding protein
75171	75524	+	RTX toxin activating lysine-acyltransferase (EC 2.3.1.-)
75552	87755	+	RTX toxins determinant A and related Ca2+-binding proteins
88400	100636	+	RTX toxins determinant A and related Ca2+-binding proteins
59707	61428	+	toxin protein
99997	100206	+	Prevent host death protein, Phd antitoxin
121847	126595	+	Putative toxin subunit
128840	132292	+	Unknown, probable insecticidal toxin
132314	136450	+	Unknown, probable insecticidal toxin
219949	219623	-	HigA protein (antitoxin to HigB)
327897	327748	-	ParE toxin protein
403153	402863	-	YefM protein (antitoxin to YoeB)
539091	539501	+	Unknown, hypothetical toxin
543217	543396	+	FIG022160: hypothetical toxin
543369	543731	+	FIG045511: hypothetical antitoxin (to FIG022160: hypothetical toxin)
38357	41632	+	Putative toxin subunit
41654	45229	+	Putative toxin subunit
45292	49749	+	Unknown, probable insecticidal toxin
51423	49831	-	Unknown, probable insecticidal toxin
50632	50345	-	HigA protein (antitoxin to HigB)
50910	50632	-	HigB toxin protein
8724	2047	-	Unknown, probable insecticidal toxin
9089	11836	+	Putative insecticidal toxin complex
65585	65800	+	Antitoxin YgiT
425121	424705	-	Antitoxin 1
1599	1841	+	VapB protein (antitoxin to VapC)
1834	2238	+	VapC toxin protein
13294	2657	-	RTX toxins determinant A and related Ca2+-binding proteins
24483	13918	-	RTX toxins determinant A and related Ca2+-binding proteins
17069	14913	-	Putative toxin transport protein
20571	18457	-	RTX toxin transporter
65989	65717	-	Prevent host death protein, Phd antitoxin
212084	207210	-	Unknown, probable toxin
158066	156807	-	Unknown, hypothetical insectidal toxins.
307869	304738	-	Putative insecticidal toxin complex
312698	308004	-	Unknown, probable insecticidal toxin
315699	312799	-	Unknown, probable insecticidal toxin
331160	334069	+	Putative insecticidal toxin complex
1923	1564	-	Programmed cell death toxin MazF
2171	1923	-	Programmed cell death antitoxin MazE
23829	24095	+	Prevent host death protein, Phd antitoxin

239286	239753	+	Prevent host death protein, Phd antitoxin
239750	240040	+	Death on curing protein, Doc toxin
70558	70307	-	VapB protein (antitoxin to VapC)
112945	112619	-	Death on curing protein, Doc toxin
113220	112945	-	Prevent host death protein, Phd antitoxin
188284	187910	-	Programmed cell death toxin PemK
221072	218265	-	Putative insecticidal toxin complex
28788	21271	-	Unknown, probable insecticidal toxin
56195	56380	+	ParD protein (antitoxin to ParE)
56373	56657	+	ParE toxin protein
25086	25982	+	dermonecrotic toxin
26203	27135	+	Putative insecticidal toxin complex
78538	82074	+	Unknown, probable insecticidal toxin
82067	86161	+	Unknown, probable insecticidal toxin
122306	117405	-	Dermonecrotic toxin
127038	124243	-	Putative insecticidal toxin complex
150818	151063	+	Programmed cell death antitoxin MazE
151063	151395	+	Programmed cell death toxin MazF
199320	192100	-	Unknown, probable insecticidal toxin
120978	123824	+	Putative insecticidal toxin complex
130138	136761	+	Unknown, probable insecticidal toxin
137080	145002	+	Unknown, probable insecticidal toxin
145059	149492	+	Unknown, probable insecticidal toxin
150029	152911	+	Putative insecticidal toxin complex
154542	162116	+	Unknown, probable insecticidal toxin
162269	165061	+	Putative insecticidal toxin complex
166838	174412	+	Unknown, probable insecticidal toxin
174469	178914	+	Unknown, probable insecticidal toxin
53727	52549	-	IncI1 plasmid conjugative transfer pilus-tip adhesin protein PilV
89270	91750	+	Unknown, probable export and assembly of fimbrial adhesin
93007	94017	+	Fimbrial adhesin precursor
44	859	+	Putative large exoprotein involved in heme utilization or adhesion of ShlA/HecA/FhaA family
97046	97711	+	Lipoprotein NlpE involeved in surface adhesion
325511	328882	+	Galactophilic lectin PA-I
139940	140110	+	Fimbrial operon regulator
93007	94017	+	Fimbrial adhesion precursor
949909	95484	+	21 kDa hemolysin precursor
226726	226267	-	Hemolysin
5492	6847	+	Siderophore biosynthesis non-ribosomal peptide synthetase modules
30584	31564	+	Iron siderophore receptor protein

Table S2. Proteins involved in the pathogenesis *Pseudomonas aeruginosa* NA04.

Start	Stop	Strand	Function
80281	82347	+	Pathogenesis related protein
573998	574381	+	PE-PGRS virulence associated protein
675441	676106	+	Virulence factor mviM
44725	44444	-	ParE toxin protein
44949	44722	-	Prevent host death protein, Phd antitoxin

insects MDPI

8852	9898	+	RTX toxins and related Ca2+-binding proteins
28549	28899	+	Accessory cholera enterotoxin
28901	30163	+	Zona occludens toxin
5934	7367	+	Multidrug and toxin extrusion (MATE) family efflux pump YdhE/NorM
511530	509614	-	exotoxin A precursor
202704	202297	-	ParE toxin protein
9900	10919	+	Large exoproteins involved in heme utilization or adhesion
55407	54460	+	Sigma-fimbriae tip adhesin
104465	104947	+	Fimbrial protein precursor
105119	105781	+	Putative fimbrial chaperone
57776	55404	-	Sigma-fimbriae usher protein
58581	57793	-	Sigma-fimbriae chaperone protein
235021	236721	+	Type IV fimbrial assembly, ATPase PilB
236725	237942	+	Type IV fimbrial assembly protein PilC
6874	687116	-	Type III effector HopPmaJ
209217	208786	-	Type III secretion effector protein YopR
331105	332601	-	Vibriolysin, extracellular zinc protease
487642	485711	-	Phospholipase/lecithinase/hemolysin
42578	43333	+	Putative hemolysin
124307	124885	+	21 kDa hemolysin precursor
59860	61938	+	Non-hemolytic phospholipase C precursor
219737	218715	-	Iron siderophore sensor protein
12	305	+	Non-ribosomal peptide synthetase modules, pyoverdine @ Siderophore biosynthesis
